# Trends in body mass index in the pre-dolutegravir period in South Africa

**DOI:** 10.4102/sajhivmed.v25i1.1523

**Published:** 2024-04-23

**Authors:** Florian van Ginkel, Roos E. Barth, Hugo Tempelman, Kerstin Klipstein-Grobusch, Diederick E. Grobbee, Karine Scheuermaier, Francois W.D. Venter, Alinda G. Vos-Seda

**Affiliations:** 1 Julius Global Health, Julius Center for Health Sciences and Primary Care, University Medical Center Utrecht, Utrecht, the Netherlands; 2 Department of Infectious Disease, University Medical Center Utrecht, Utrecht University, Utrecht, the Netherlands; 3 Ndlovu Care Group, Groblersdal, South Africa; 4 Division of Epidemiology and Biostatistics, School of Public Health, Faculty of Health Sciences, University of the Witwatersrand, Johannesburg, South Africa; 5 School of Physiology, Faculty of Health Sciences, University of the Witwatersrand, Johannesburg, South Africa; 6 Department of Ezintsha, Faculty of Health Sciences, University of the Witwatersrand, Johannesburg, South Africa

**Keywords:** body mass index, obesity, sub-Saharan Africa, HIV, antiretroviral therapy, integrase strand transfer inhibitors

## Abstract

**Background:**

Antiretroviral therapy (ART) is associated with weight gain, but this has been shown to be more marked with dolutegravir and other integrase strand transfer inhibitors.

**Objectives:**

We studied weight gain in people living with HIV (PLWH) on ART compared to the general population in the period before dolutegravir was introduced in a rural South African cohort.

**Method:**

Longitudinal analysis of the Ndlovu Cohort Study including 36–48 months’ follow-up data. From 2014 to 2019, data were collected annually in Limpopo, rural South Africa. Linear mixed models using HIV status, demographics, ART use and cardiovascular risk factors were used to estimate trends in body mass index (BMI) over time.

**Results:**

In total, 1518 adult, non-pregnant participants were included, of whom 518 were PLWH on ART (79.8%), 135 PLWH not yet on ART (20.2%) and 865 HIV-negative. HIV-negative participants had significantly higher BMIs than PLWH on ART at all study visits. There was a significant increase in BMI in all subgroups after 36 months (PLWH on ART, BMI +1.2 kg/m^2^, *P* < 0.001; PLWH not on ART, BMI +1.8 kg/m^2^, *P* < 0.001 and HIV-negative, BMI +1.3 kg/m^2^, *P* < 0.001).

**Conclusion:**

The increase in BMI in PLWH and HIV-negative participants is a serious warning signal as obesity results in morbidity and mortality.

**What this study adds:** Weight gain is a concern in HIV-positive people using integrase strand transfer inhibitors (INSTIs). This study shows that there was significant weight gain in both HIV-positive and HIV-negative people before the introduction of INSTIs, indicating that weight gain is a population-wide problem necessitating health interventions.

## Introduction

The world is facing an obesity epidemic. Since 1980, the prevalence of obesity has doubled in more than 70 countries, resulting in approximately 600 million adult people with obesity in 2015.^1^ Overweight and obesity are risk factors for diabetes mellitus and cardiovascular diseases and result in an increased risk of premature mortality.^2,3^ Not only high-income countries suffer from this global pandemic. Trends in rising obesity rates in low- and middle-income countries have been reported as well.^4^ In 2016, 31% of men older than 15 years were overweight or obese in South Africa, and 68% of women.^5^

The obesity epidemic intersects with the HIV epidemic. At the end of 2019, 38 million people were living with an HIV infection globally.^6^ The most severely affected region is sub-Saharan Africa (SSA), where about two-thirds of all people living with HIV (PLWH) reside.^6^ Since the introduction of combined antiretroviral therapy (ART) in 1996, HIV-related morbidity and mortality has decreased and life expectancy increased.^7^ Together with an increased life expectancy, the risk of age- and lifestyle-related comorbidities, including obesity, also increased.^8^

In 2018, the World Health Organization recommended to change first-line ART to an integrase strand transfer inhibitor (INSTI)-based regimen.^9^ The introduction of dolutegravir and other INSTIs has been associated with greater weight gain than non-INSTI regimens.^10,11^ Since then, the scientific spotlight has been on INSTI-related weight gain. Less is known about weight gain on previous first-line ART regimens, especially with extended use. Furthermore, little is known about weight gain in PLWH ART in the pre-2018 period compared to weight gain in the HIV-negative population. This study aims to gain insight into weight gain in PLWH on ART or initiating ART compared to weight gain in the HIV-negative population in South Africa in the pre-INSTI period over a period of 36–48 months.

## Methods

This study is a secondary data analysis of the Ndlovu Cohort study (NCS). The NCS is located in Limpopo province, South Africa and was set up to investigate the influence of HIV and ART on cardiovascular risk factors and cardiovascular events in a rural African population. The design and methods have been described previously.^12^ In short, inclusion criteria were being aged 18 years and older, being able to provide written informed consent, and being committed to long-term follow-up. Study enrolment took place with the help of community campaigns, and participants were recruited at local events, shopping centres and at the Ndlovu Medical Centre (NMC). The NMC included a Department of Health-contracted HIV treatment facility serving approximately 3700 PLWH. Upon enrolment in the NCS, participants underwent HIV testing unless they were on HIV treatment. First-line and second-line ART were defined according to the South African National Department of Health guidelines.^13^ ART treatment status at baseline was assessed by self-report and complemented with data from an electronic HIV registry (TIER.net). ART treatment during follow up was assessed by self-report. Study approval was obtained from the Human Research Ethics Committee of the University of Pretoria, South Africa, and the Limpopo Department of Health Ethics Committee. Written informed consent was obtained from all participants prior to study participation.

### Measurements and definitions

Data were collected on demographics, medical history and medication use using standardised questionnaires. Blood samples were drawn to measure CD4 cell count and viral load (VL). All participants were invited for annual follow-up visits for up to 48 months, during which anthropometric measurements, including height and weight, CD4 cell count and VL were measured again. The same scales were used during the survey. Body mass index (BMI, kg/m^2^) was calculated using height at baseline. BMI was classified as ‘underweight’ (BMI < 18.5 kg/m^2^), ‘normal weight’ (BMI 18.5–24.9 kg/m^2^), ‘overweight’ (BMI 25.0–29.9 kg/m^2^) or ‘obese’ (BMI ≥ 30.0 kg/m^2^). Intake of vegetables and fruit, used as a proxy for healthy food, was categorised as: ‘poor’ (< 2 servings/day), ‘intermediate’ (2–4 servings/day) or ‘good’ (≥ 5 servings/day). Monthly income was categorised in three categories: less than R648.00 (South African rand) (≈$46.00) (below the poverty line), between R648.00 and R992.00, and more than R992.00, as defined by Statistics South Africa in the period of study enrolment.^14^ Employment status was defined as: ‘unemployed’, ‘self-employed’ or ‘other’ (student, retired, volunteer). Relationship status was defined as ‘stable’, including married, cohabiting or having a life partner, or ‘unstable’, including divorced, single, widowed or multiple partners. The highest level of education level was categorised as ‘none’, ‘primary’, ‘secondary and matric’ and ‘college and university’. Physical activity levels were measured with the International Physical Activity Questionnaire and categorised as ‘low’, ‘moderate’ or ‘high’.^15,16^

### Statistical analysis

Demographics were reported as mean and standard deviation, median with interquartile range or count with percentage, as appropriate. At baseline, participants were divided in three groups: ‘PLWH on ART’, ‘PLWH not on ART’, and ‘HIV-negative’. Differences in demographics and clinical characteristics between groups at baseline was presented using descriptive statistics. Participants on first- and second-line ART were combined, since the percentage of participants on second-line ART was too small to be analysed separately. We excluded participants with missing data on ART at baseline, participants with only a single visit, and female participants who reported themselves to be pregnant at any visit.

The trend in BMI over time was analysed with linear mixed models (estimated with maximum likelihood). We used two different approaches to categorise patients, both aiming to calculate estimated marginal means for BMI by HIV and ART status. In the first approach, assignment to a group was flexible over time, for example depending on HIV and ART status at a specific visit. Participants could possibly, therefore, change groups every follow-up visit. ART use was defined in two different ways. In model 1, HIV status and self-reported ART status was used to define the following three groups: ‘PLWH on ART’, ‘PLWH not on ART’ and ‘HIV-negative’. In model 2, VL was used as a proxy for ART use. A VL < 1000 copies/mL was classified as ‘PLWH on ART’, a VL ≥ 1000 copies/mL as ‘PLWH not on ART’.

In the second approach (model 3), assignment to a group was fixed. Participants were assigned to a group according to HIV status and VL at baseline and follow-up, so participants could not vary between groups during follow-up. PLWH on ART with VL < 1000 copies/mL at baseline, who remained virally suppressed (VL < 1000 copies/mL) during follow-up, were classified as ‘PLWH on stable ART’. At baseline, PLWH who were virally unsuppressed (VL ≥ 1000 copies/mL) (regardless of ART use) with viral suppression during all attended follow-up visits were classified as ‘PLWH initiating ART’. Participants with either VL < 1000 copies/mL or VL ≥ 1000 copies/mL at baseline and at least one follow-up visit with VL ≥ 1000 copies/mL were categorised as ‘PLWH either ART non-adherence or therapy resistance’. Participants who were HIV-negative at study enrolment but tested HIV-positive at any follow-up visit were marked as ‘seroconverters’, and participants who were negative at study enrolment and remained HIV-negative, as ‘HIV-negative’. In this approach, participants needed to have at least one available VL result during follow-up to be included.

In all three models, we used a random intercept and a random effect for time. The following variables were included as fixed effects: HIV treatment status (as defined per model), sex, age, time, time on ART at baseline, income per month, relationship status, physical activity, educational level, fruit and vegetable intake, smoking status, and the interaction between time and both HIV treatment status and gender. Time on ART at baseline was set to zero for participants not on ART at study enrolment. Known duration of HIV infection was not included in our model, as a high correlation with ART duration was expected. To avoid assumptions of the BMI trend over time, BMI was included as both an ordinal and a continuous variable. Results are presented as estimated marginal means and regression coefficients (β) with 95% confidence intervals.

Statistical testing was limited to 36 months’ follow-up data, although 48-month follow-up data are included in the figures. Physical visits ended in 2019 due to budget constraints and not all participants had completed a 48-month study visit. The majority of HIV-positive participants were included in the second half of the inclusion period as baseline. Consequently, more HIV-positive than HIV-negative participants missed the 48-month visit, and missing at random cannot be guaranteed. A *P*-value ≤ 0.05 was considered to be statistically significant. Statistical analysis was done with Statistical Package for Social Sciences version 26 (IBM SPSS Statistics for Windows, Version 26.0. Armonk, New York: IBM Corp).

### Ethical considerations

Ethical clearance to conduct this study was obtained from the University of Pretoria, Faculty of Health Sciences Research Ethics Committee (No. 227/2014).

## Results

The NCS included 1927 participants. We excluded three participants due to missing ART information at baseline, 342 participants without any follow-up visit and 64 female participants due to pregnancy at any point in the study. In total, 1518 participants were included (Table 1). Mean age of study participants was 39.4 years (standard deviation [s.d.]: 12.9), the majority were female (817 women; 54.1%, *P* < 0.001). In total, 518 PLWH (79.3%) were on ART at study enrolment, 135 PLWH (20.7%) were not yet on ART and 865 participants were HIV-negative. In total, 463 participants (89.4%) were on first-line ART and 55 participants (10.6%) on second-line ART. PLWH on ART were significantly older compared to HIV-negative participants (43.2 years versus 37.3 years, *P* < 0.001). BMI of HIV-negative participants at baseline was higher compared to PLWH on ART (24.7 kg/m^2^ versus 23.6 kg/m^2^, *P* = 0.009). Female participants had a significantly higher BMI compared to male participants (26.4 kg/m^2^ versus 21.7 kg/m^2^, *P* < 0.001). Most study participants had a normal weight (726 participants, 47.8%). Median time since HIV diagnosis for PLWH on ART was 72.0 months (interquartile range [IQR]: 33.5–107.0). During follow-up, 32 participants were newly diagnosed with HIV (median time to seroconversion was 23.0 months, IQR: 12.3–35.0). Up to the 36-month follow-up timepoint, 5196 follow-up visits with available weight, and hence BMI, were included. At 36 months, loss to follow-up was 25.7% (at 48 months, loss to follow-up was 62.7%). The distribution of population characteristics at 36 months did not differ from the distribution of these characteristics at baseline (Table 2). On average, all groups, regardless of HIV and ART status, gained weight during study follow-up (Figure 1). Figure 2 shows the increase in percentage of PLWH on ART with overweight during study follow-up (18.9% at baseline versus 25.9% at 36 months) and obesity (13.5% at baseline versus 20.5% at 36 months). For HIV-negative participants, the percentage of overweight people did not change much (21.4% at baseline and 21.6% at 36 months), but obesity increased from 19.8% at baseline to 26.1% at 36 months.

**FIGURE 1:**
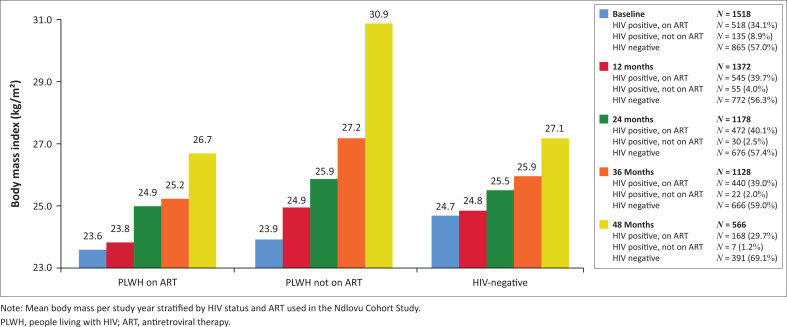
Body mass index over time in the Ndlovu Cohort Study.

**FIGURE 2:**
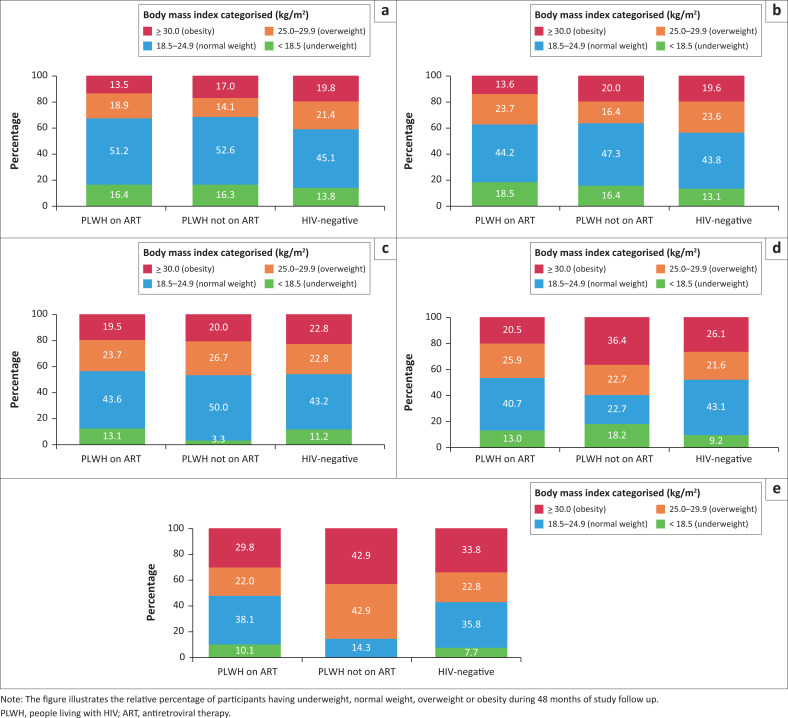
Relative percentages of body mass index categorised at baseline and after 48 months of study follow up in Ndlovu Cohort Study. (a) Baseline (*N* = 1518); (b) 12 months (*N* = 1372); (c) 24 months (*N* = 1178); (d) 36 months (*N* = 1128) and (e) 48 months (*N* = 566).

**TABLE 1:** Distribution of population characteristics at baseline.

Variables	PLWH on ART (*n* = 518)						PLWH not on ART (*n* = 135)						HIV-negative (*n* = 865)						All participants (*N* = 1518)					
	*n*	%	Median	IQR	Mean	s.d.	*n*	%	Median	IQR	Mean	s.d.	*n*	%	Median	IQR	Mean	s.d.	*n*	%	Median	IQR	Mean	s.d.
First-line ART	463	89.4	-	-	-	-	NA	-	-	-	-	-	NA	-	-	-	-	-	463	30.5	-	-	-	-
Second-line ART	55	10.6	-	-	-	-	-	-	-	-	-	-	-	-	-	-	-	-	55	3.6	-	-	-	-
Age (years)		-	-	-	43.2	9.7		-	-	-	38.6	11.0		-	-	-	37.3	14.3		-	-	-	39.4	12.9
Women	310	59.8	-	-	-	-	82	60.7	-	-	-	-	425	49.1	-	-	-	-	817	53.8	-	-	-	-
**Highest level of education**																								
None	24	4.6	-	-	-	-	4	3.0	-	-	-	-	38	4.4	-	-	-	-	66	4.3	-	-	-	-
Primary	125	24.1	-	-	-	-	37	27.4	-	-	-	-	151	17.5	-	-	-	-	313	20.6	-	-	-	-
Secondary and matric	337	65.1	-	-	-	-	82	60.7	-	-	-	-	586	67.7	-	-	-	-	1005	66.2	-	-	-	-
College and university	32	6.2	-	-	-	-	12	8.9	-	-	-	-	90	10.4	-	-	-	-	134	8.8	-	-	-	-
**Employment status**																								
Unemployed	348	67.2	-	-	-	-	100	74.1	-	-	-	-	582	67.3	-	-	-	-	1030	67.9	-	-	-	-
Employed	152	29.3	-	-	-	-	27	20.0	-	-	-	-	134	15.5	-	-	-	-	313	20.6	-	-	-	-
Other (student, retired, volunteer)	18	3.5	-	-	-	-	8	5.9	-	-	-	-	149	17.2	-	-	-	-	175	11.5	-	-	-	-
**Income per month (ZAR)† (*N* = 1439)**																								
< 648	305	62.0	-	-	-	-	86	67.2	-	-	-	-	516	63.0	-	-	-	-	907	63.0	-	-	-	-
648–992	41	8.3	-	-	-	-	11	8.6	-	-	-	-	67	8.2	-	-	-	-	119	8.3	-	-	-	-
> 992	146	29.7	-	-	-	-	31	24.2	-	-	-	-	236	28.8	-	-	-	-	413	28.7	-	-	-	-
Stable relationship (married, life partner, cohabiting)	283	54.6	-	-	-	-	62	45.9	-	-	-	-	527	60.9	-	-	-	-	872	57.4	-	-	-	-
**Smoking**																								
Ever (*N* = 1499)	181	35.7	-	-	-	-	61	47.7	-	-	-	-	385	44.6	-	-	-	-	627	41.8	-	-	-	-
Current	121	23.4	-	-	-	-	47	34.8	-	-	-	-	283	32.7	-	-	-	-	451	29.7	-	-	-	-
Cigarettes/cigars per day	-	-	5.5	4.0 – 10.0	-	-	-	-	6.0	5.0 – 12.0	-	-	-	-	6.0	4.0 – 10.0	-	-	-	-	6.0	4.0 – 10.0	-	-
**Physical activity**																								
Moderate	166	32.0	-	-	-	-	48	35.6	-	-	-	-	337	39.0	-	-	-	-	551	36.3	-	-	-	-
High	115	22.2	-	-	-	-	39	28.9	-	-	-	-	244	28.2	-	-	-	-	398	26.2	-	-	-	-
**Fruit and vegetable consumption**																								
Poor (< 2 servings/day)	267	51.5	-	-	-	-	84	62.2	-	-	-	-	543	62.8	-	-	-	-	894	58.9	-	-	-	-
Intermediate (2–4 servings/day)	220	42.5	-	-	-	-	40	29.6	-	-	-	-	292	33.8	-	-	-	-	552	36.4	-	-	-	-
Good (≥ 5 servings/day)	31	6.0	-	-	-	-	11	8.1	-	-	-	-	30	3.5	-	-	-	-	72	4.7	-	-	-	-
BMI (kg/m^2^)	-	-	-	-	23.6	5.7	-	-	-	-	23.9	6.4	-	-	-	-	24.7	6.2	-	-	-	-	24.2	6.1
Women	-	-	-	-	25.2	6.0	-	-	-	-	26.1	6.9	-	-	-	-	27.3	6.8	-	-	-	-	26.4	6.6
Men	-	-	-	-	21.2	4.0	-	-	-	-	20.4	3.6	-	-	-	-	22.1	4.3	-	-	-	-	21.7	4.2
BMI < 18.5 (underweight)	85	16.4	-	-	-	-	22	16.3	-	-	-	-	119	13.8	-	-	-	-	226	14.9	-	-	-	-
BMI 18.5–24.9 (normal weight)	265	51.2	-	-	-	-	71	52.6	-	-	-	-	390	45.1	-	-	-	-	726	47.8	-	-	-	-
BMI 25.0–30.0 (overweight)	98	18.9	-	-	-	-	19	14.1	-	-	-	-	185	21.4	-	-	-	-	302	19.9	-	-	-	-
BMI ≥ 30.0 (obesity)	70	13.5	-	-	-	-	23	17.0	-	-	-	-	171	19.8	-	-	-	-	264	17.4	-	-	-	-
**HIV-related characteristics**																								
Time in months since HIV diagnosis‡	-	-	72.0	33.5 – 107.0	-	-	-	-	0.0	0.0 – 11.5	-	-	NA	-	-	-	-	-	-	-	59.0	14.0 – 101.0	-	-
Time in months on ART	-	-	61.0	23.5 – 99.5	-	-	-	-	0.0	0.0 – 0.0	-	-	NA	-	-	-	-	-	-	-	45.0	4.8 – 93.0	-	-
CD4+ cell count, cells/mm^3^	-	-	504.0	351.0 – 677.5	-	-	-	-	393.0	279.5 – 565.5	-	-	NA	-	-	-	-	-	-	-	486.0	335.0 – 660.5	-	-
CD4+ < 200, cells/mm^3^	39	7.6	-	-	-	-	23	17.4	-	-	-	-	NA	-	-	-	-	-	62	4.1	-	-	-	-

PLWH, people living with HIV; ART, antiretroviral therapy; NA, non-applicable; IQR, interquartile range; s.d., standard deviation; ZAR, South African Rand; BMI, body mass index.

† , Income per person per month. Lower-bound poverty line: < 648, upper-bound poverty line > 991.

‡ , Time since conversion to HIV at study enrolment.

**TABLE 2:** Distribution of population characteristics at 36 months follow up.

Variables	PLWH on ART (*N* = 440)						PLWH not on ART (*N* = 22)						HIV-negative (*N* = 666)						All participants (*N* = 1128)					
	*n*	%	Median	IQR	Mean	s.d.	*n*	%	Median	IQR	Mean	s.d.	*n*	%	Median	IQR	Mean	s.d.	*n*	%	Median	IQR	Mean	s.d.
**Age (years)**	-	-	-	-	41.7	9.8	-	-	-	-	34.4	11.0	-	-	-	-	38.2	14.4	-	-	-	-	39.5	12.8
Women	289	65.7	-	-	-	-	16	72.7	-	-	-	-	321	48.2	-	-	-	-	626	55.5	-	-	-	-
**Highest level of education**																								
None	16	3.6	-	-	-	-	0	0.0	-	-	-	-	31	4.7	-	-	-	-	47	4.2	-	-	-	-
Primary	107	24.3	-	-	-	-	3	13.6	-	-	-	-	123	18.5	-	-	-	-	233	20.7	-	-	-	-
Secondary and matric	289	65.7	-	-	-	-	14	63.6	-	-	-	-	446	67.0	-	-	-	-	749	66.4	-	-	-	-
College and university	28	6.4	-	-	-	-	5	22.7	-	-	-	-	66	9.9	-	-	-	-	99	8.8	-	-	-	-
**Employment status**																								
Unemployed	308	70.0	-	-	-	-	18	81.8	-	-	-	-	442	66.4	-	-	-	-	768	68.1	-	-	-	-
Employed	113	25.7	-	-	-	-	3	13.6	-	-	-	-	112	16.8	-	-	-	-	228	20.2	-	-	-	-
Other (student, retired, volunteer)	19	4.3	-	-	-	-	1	4.5	-	-	-	-	112	16.8	-	-	-	-	132	11.7	-	-	-	-
**Income per month (ZAR)† (*N* = 1075)**																								
< 648	272	64.5	-	-	-	-	15	68.2	-	-	-	-	397	62.9	-	-	-	-	684	63.6	-	-	-	-
648–992	38	9.0	-	-	-	-	1	4.5	-	-	-	-	50	7.9	-	-	-	-	89	8.3	-	-	-	-
> 992	112	26.5	-	-	-	-	6	27.3	-	-	-	-	184	29.2	-	-	-	-	302	28.1	-	-	-	-
Stable relationship (married, life partner, cohabiting)	229	52.0	-	-	-	-	11	50.0	-	-	-	-	410	61.6	-	-	-	-	650	57.6	-	-	-	-
**Smoking**																								
Ever (*N* = 1121)	153	35.3	-	-	-	-	9	40.9	-	-	-	-	304	45.6	-	-	-	-	466	41.6	-	-	-	-
Current	106	24.1	-	-	-	-	8	36.4	-	-	-	-	219	32.9	-	-	-	-	333	29.5	-	-	-	-
Cigarettes/cigars per day	-	-	6.0	3.0–10.0	-	-	-	-	4.0	3.0–6.0	-	-	-	-	6.0	4.0–10.0	-	-	-	-	6.0	4.0–10.0	-	-
**Physical activity**																								
Moderate	156	35.5	-	-	-	-	13	59.1	-	-	-	-	253	38.0	-	-	-	-	422	37.4	-	-	-	-
High	101	23.0	-	-	-	-	3	13.6	-	-	-	-	188	28.2	-	-	-	-	292	25.9	-	-	-	-
**Fruit and vegetable consumption**																								
Poor (< 2 servings/day)	247	56.1	-	-	-	-	13	59.1	-	-	-	-	424	63.7	-	-	-	-	684	60.6	-	-	-	-
Intermediate (2–4 servings/day)	164	37.3	-	-	-	-	8	36.4	-	-	-	-	221	33.2	-	-	-	-	393	34.8	-	-	-	-
Good (≥ 5 servings/day)	29	6.6	-	-	-	-	1	4.5	-	-	-	-	21	3.2	-	-	-	-	51	4.5	-	-	-	-

Note: Lower-bound poverty line: < 648, upper-bound poverty line > 991.

PLWH, people living with HIV; ART, antiretroviral therapy; NA, not applicable; s.d., standard deviation; IQR, interquartile range; ZAR, South African Rand.

† , Income per person per month.

**FIGURE 3:**
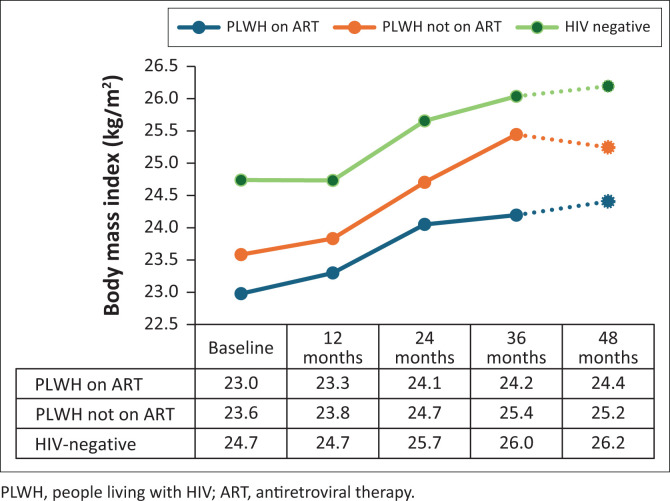
Body mass index over time by self-reported antiretroviral therapy (model 1).

Model 1 (Figure 3) shows the trends in BMI over time, where groups are defined using self-reported ART status. All subgroups had a significant weight gain at 36 months compared to the baseline visit (PLWH on ART, BMI +1.2 kg/m^2^, *P* < 0.001; PLWH not on ART, BMI +1.9 kg/m^2^, *P* < 0.001; HIV-negative, BMI +1.3 kg/m^2^, *P* < 0.001). Weight gain was significant in the first 12 months in PLWH on ART (BMI +0.3 kg/m^2^, *P* = 0.001). HIV-negative participants had a significantly higher BMI compared to PLWH on ART at all study visits (after 36 months BMI +1.8 kg/m^2^, *P* < 0.001). Model 2 (Figure 1-A1), using VL as a proxy for ART use, showed similar trends in BMI compared to model 1.

In model 3 (Figure 2-A1), participants had a fixed allocation to one group. In total, 1499 participants were included in this analysis (411 PLWH on stable ART, 96 PLWH initiating HIV, 127 PLWH either ART non-adherence or therapy resistant, 32 seroconverters and 833 HIV-negative participants). PLWH on stable ART, PLWH initiating ART and HIV-negative individuals had significant weight gain from baseline to 36 months (PLWH on stable ART, BMI +1.3 kg/m^2^, *P* < 0.001; PLWH initiating ART, BMI +1.0 kg/m^2^, *P* = 0.001; HIV-negative; BMI +1.3 kg/m^2^, *P* < 0.001). PLWH either ART non-adherence or therapy resistant (BMI +0.3 kg/m^2^, *P* = 0.229) and seroconverters (BMI +0.7 kg/m^2^, *P* = 0.147) had no significant weight gain in 36 months. In the first 12 months, weight gain was most pronounced in PLWH who initiated ART (BMI +0.5 kg/m^2^, *P* = 0.026), but also increased in PLWH on stable ART (BMI +0.4 kg/m^2^, *P* < 0.001). From 12 months to 36 months, weight continued to increase but the trend in BMI was attenuated in PLWH compared to HIV-negative participants, for whom BMI increased significantly between every visit (12–24 months, BMI +0.9 kg/m^2^, *P* < 0.001 and 24–36 months, BMI +0.4 kg/m^2^, *P* < 0.001). At every study visit, except for the 12 months visit, HIV-negative participants were significantly heavier than PLWH on stable ART and PLWH initiating ART (after 36 months, HIV-negative participants versus PLWH on stable ART, BMI +1.7 kg/m^2^, *P* = 0.007, and HIV-negative participants versus PLWH initiating ART, BMI +2.2 kg/m^2^, *P* = 0.012).

In all models, female gender, higher educational status (university and college), currently not smoking, fruit and vegetable intake as dietary proxy and age were significantly associated with a higher BMI (*P* < 0.05). The initial BMI was inversely correlated with the duration of ART treatment (*P* < 0.05) (Appendix 1, Table 1-A1).

## Discussion

Weight-gain trajectories in PLWH on or initiating ART compared to HIV-negative participants were similar and significant. This suggests that the weight gain seen in various observational studies and randomised controlled trials (RCTs) is, at least in part, a return to an obesogenic population trajectory. This observation aligns with the findings of other observational studies, affirming a sustained increase in BMI over time in patients, regardless of their HIV status.^17,18,19^

There is ample evidence that PLWH starting INSTI gain significantly more weight compared to PLWH using a first-line ART regimen without INSTI.^10,20,21,22^ However, most studies addressing weight gain in people on INSTI-based ART lack comparison with HIV-negative controls, which makes it hard to address excess weight gain compared to the non-HIV-positive population. RCTs conducted in ART-naïve PLWH in Johannesburg, South Africa, and in Yaoundé, Cameroon, showed a significant increase in weight over 96 weeks in people on a dolutegravir-emtricitabine-tenofovir-containing regimen compared to the standard care group receiving a tenofovir disoproxil fumarate and efavirenz-based regimen.^22,23^ The trend in weight gain observed in people on non-INSTI regiments during 96 weeks follow-up in the South African RCT is comparable to our study results (at 96 weeks, participants on non-INSTI containing ART increased 2.3 kg versus 2.4 kg in PLWH initiating ART in our study after 24 months).^23^ The Swiss Cohort study, an observational study between 1990 and 2012 with 1601 PLWH (80% male, predominantly men who have sex with men), found that BMI increased most steeply within the first year of ART use (BMI +0.92 kg/m^2^, 95% CI: 0.8–1.0 kg/m^2^) whereafter BMI continued to increase, but at a lower rate (BMI +0.31 kg/m^2^ per year, 95% CI: 0.29–0.34 kg/m^2^).^24^ The steep increase in weight in the first 12 months is in line with what we found in PLWH initiating ART (BMI +0.52 kg/m^2^ per year, 95% CI: 0.06 – 0.98 kg/m^2^), but in our study the curve did not flatten after 12 months, unlike in the Swiss Cohort study. The continued increase in BMI observed in our study could be related to the obesogenic environment, differences in population demographics such as ethnicity and gender, as well as age-related increase in BMI.^25^ The North American AIDS Cohort Collaboration on Research and Design study was another observational study which analysed BMI of 14 084 PLWH (83% male, 57% non-Caucasian) between 1998 and 2010.^26^ Trends in BMI were compared to BMI trends in the general United States population. During the study period, the prevalence of obesity increased more in PLWH on ART compared to HIV-negative participants, although weight increased in both groups (in 1998, prevalence of obesity was 9% in PLWH on ART versus 22% in HIV-negative participants and in 2010, 18% of PLWH on ART were obese versus 27% controls). In line with these findings, we found an increased percentage in obesity rates in both PLWH on ART and HIV-negative participants over time, namely a 7.0% increase in obesity (from 13.5% to 20.5%) after 36 months in PLWH on ART and 6.3% increase in obesity (from 19.8% to 26.1%) in HIV-negative participants after the 36-month follow-up.

In our second model (Figure 1-A1), VL was used as a proxy for ART use to account for therapy non-adherence and ART failure. As the trends in BMI were similar to the trends seen in model 1 (Figure 3), the use of self-reported ART information seems to be reliable.

Although we did not find excessive weight gain in PLWH compared to HIV-negative participants, the increase in BMI in general was significant. As the prevalence of obesity is rising, the risk of comorbidities like cardiovascular diseases, type 2 diabetes mellitus, hypertension, sleep apnoea and some malignancies is increasing.^27^ As a result, overweight or obese people have an increased risk of all-cause mortality.^28^

Weight gain in our study was most pronounced in women, regardless of HIV status. Other studies on PLWH, including studies addressing INSTI-based ART regiments, also observed excessive weight gain in women compared to men.^22,23,29,30,31^ In HIV-negative women in South Africa, poverty in childhood and the lack of access to resources in adulthood life are associated with higher obesity rates compared to HIV-negative men.^32^ Other factors influencing the increase in body weight are urbanisation, unhealthy diets linked to availability of fast food, and poverty and social perceptions.^33^

### Strengths and limitations

Our study is the largest study in rural SSA investigating BMI over time, including both PLWH and HIV-negative controls. Another strength is the implementation of a linear mixed model, which could adequately handle missing data and hence optimise use of the data. We present data on the group ‘PLWH not yet on ART’. In line with current treatment recommendations, most participants initiated treatment upon diagnosis. As a result, the group ‘not on ART’ is based on small numbers from the first follow-up visit. This is, however, not the case in the model where ART use is defined based on VL, as that model provides insight into the group of PLWH that is either therapy non-compliant or resistant to ART. Interpretation of both models provides comprehensive insight into the effect of continued viraemia on BMI. Finally, we used models in which participants’ allocation to a group could change based on self-reported ART and VL per visit, and a model where allocation to a group was fixed over time. Therefore, our models are suitable to make both general statements about the trend in BMI over time, as well as individualised statements (e.g., the expected trajectory of a participant based on viral suppression during study follow-up). The main limitation in our study is the loss to follow-up percentage of 25.7% at 36 months. However, loss to follow-up is regarded to be missing at random, and this is supported by the finding that the distribution of population characteristics at 36 months is more or less the same as at baseline. The second limitation in our study is the self-reported ART use during follow-up. Although we used VL as proxy for ART use, more accurate data on ART use as well as information on specific ART regimens would possibly have added valuable data. A third limitation is the low prevalence of second-line ART use in our study. Therefore, we could not study the impact of different ART regimens on BMI.

## Conclusion

Pre-INSTI efavirenz-based ART regimens did not result in excessive weight gain in PLWH compared to HIV-negative participants. Among PLWH on or initiating ART, BMI increased significantly in the first 12 months. Over the course of 36 months, there was a significant increase in BMI in the whole population, similar across groups. This is a serious warning signal as obesity results in morbidity and mortality. Policymakers and healthcare workers should prioritise awareness and intervention campaigns to combat the increasing prevalence of unhealthy body weight. Future studies should focus on INSTI-related weight gain in PLWH compared to HIV-negative controls in SSA to seek out whether an increase in BMI is more significant in PLWH than in HIV-negative controls.

## Figures and Tables

**FIGURE 1-A1:**
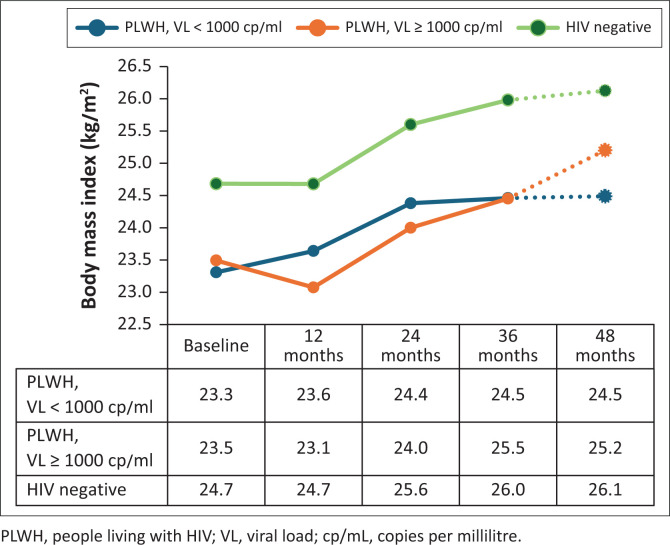
Body mass index over time by viral load suppression as proxy for antiretroviral therapy (model 2).

**FIGURE 2-A1:**
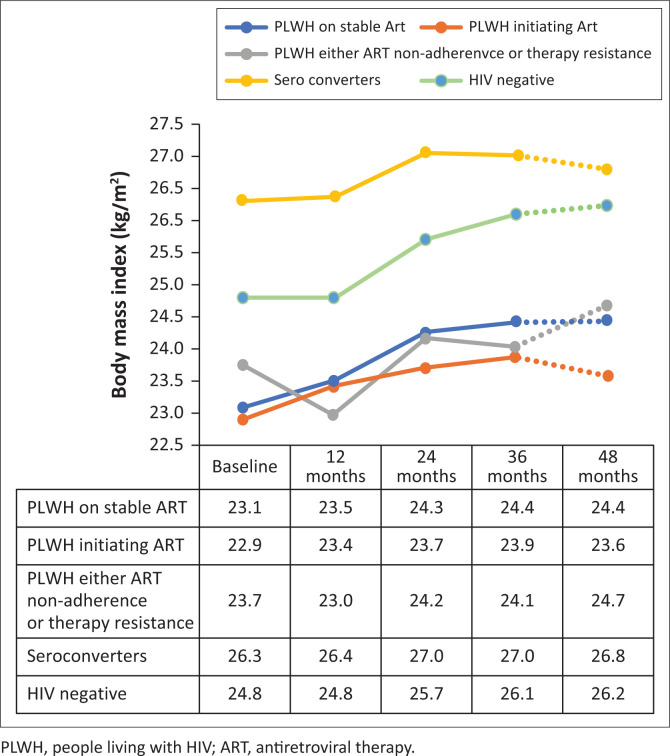
Body mass index over time stratified by fixed groups (model 3).

## Appendix 1

**TABLE 1-A1:** Estimates of fixed effects model 1, 2 and 3.

Parameters	Regression coefficient	95% CI	*P* *
**Intercept**			
Model 1	17.9	15.8 – 20.1	< 0.001
Model 2	18.0	15.9 – 20.1	< 0.001
Model 3	17.8	15.6 – 19.9	< 0.001
**Model 1**			
Time (cat) at baseline visit	REF	-	-
Time (cat) at 12 months	0.2	0.0 – 0.4	0.078
Time (cat) at 24 months	0.9	0.6 – 1.1	< 0.001
Time (cat) at 36 months	1.0	0.8 – 1.3	< 0.001
Time (cat) at 48 months	1.2	0.9 – 1.6	< 0.001
**Model 2**			
Time (cat) at baseline visit	REF	-	-
Time (cat) at 12 months	0.2	0.0 – 0.4	0.102
Time (cat) at 24 months	0.9	0.6 – 1.1	< 0.001
Time (cat) at 36 months	1.0	0.8 – 1.3	< 0.001
Time (cat) at 48 months	1.2	0.8 – 1.6	< 0.001
**Model 3**			
Time (cat) at baseline visit	REF	-	-
Time (cat) at 12 months	0.2	0.0 – 0.4	0.132
Time (cat) at 24 months	0.8	0.6 – 1.1	< 0.001
Time (cat) at 36 months	1.0	0.8 – 1.3	< 0.001
Time (cat) at 48 months	1.2	0.8 – 1.6	< 0.001
**Model 1**			
PLWH on ART	−1.8	−2.3 – −1.2	< 0.001
PLWH not on ART	−1.2	−1.8 – −0.6	< 0.001
HIV-negative	REF	-	-
**Model 2**			
PLWH, VL < 1000	−1.4	−1.9 – −0.8	< 0.001
PLWH, VL ≥ 1000	−1.2	−1.8 – −0.6	< 0.001
HIV-negative	REF	-	-
**Model 3**			
PLWH on stable ART	−1.7	−2.6 – −0.8	< 0.001
PLWH either ART non-adherence or therapy resistance	−1.1	−2.2 – 0.0	0.056
PLWH, treatment naïve	−1.9	−3.1 – −0.7	0.002
Seroconverters	1.5	−0.4 – 3.4	0.126
HIV-negative	REF	-	
**Gender**			
**Model 1**			
Female	4.0	3.3 – 4.6	< 0.001
Male	REF	-	-
**Model 2**			
Female	3.9	3.3 – 4.6	< 0.001
Male	REF	-	-
**Model 3**			
Female	4.0	3.3 – 4.6	< 0.001
Male	REF	-	-
**Income per month categorised**			
**Model 1**			
> 922 ZAR	0.4	−0.3 – 1.0	0.242
648–992 ZAR	0.4	−0.7 – 1.4	0.499
< 648 ZAR	REF	-	
**Model 2**			
> 922 ZAR	0.3	−0.3 – 1.0	0.292
648–992 ZAR	0.3	−0.7 – 1.4	0.512
< 648 ZAR	REF	-	-
**Model 3**			
> 922.00 ZAR	0.4	−0.2 – 1.1	0.183
648.00–992.00 ZAR	0.4	−0.7 – 1.4	0.496
< 648.00 ZAR	REF	-	-
**Education categorised**			
**Model 1**			
Primary	0.7	−0.7 – 2.2	0.330
Secondary & matric	1.4	−0.1 – 2.9	0.059
College & university	2.0	0.3 – 3.8	0.024
None	REF	-	-
**Model 2**			
Primary	0.7	−0.8 – 2.2	0.367
Secondary & matric	1.3	−0.1 – 2.8	0.075
College & university	2.0	0.2 – 3.8	0.025
None	REF	-	-
**Model 3**			
Primary	0.7	−0.8 – 2.2	0.359
Secondary & matric	1.3	−0.2 – 2.8	0.081
College & university	1.9	0.2 – 3.7	0.031
None	REF	-	-
**Currently smoking**			
**Model 1**			
Yes	−2.1	−2.8 – −1.4	< 0.001
No	REF	-	-
**Model 2**			
Yes	−2.1	−2.8 – −1.4	< 0.001
No	REF	-	-
**Model 3**			
Yes	−2.1	−2.8 – −1.4	< 0.001
No	REF	-	-
**IPAQ categorised**			
**Model 1**			
High	−0.6	−1.4 – 0.1	0.116
Moderate	−0.7	−1.4 – 0.1	0.035
Low	REF	-	-
**Model 2**			
High	−0.6	−1.3 – 0.2	0.130
Moderate	−0.7	−1.4 – 0.1	0.037
Low	REF	-	-
**Model 3**			
High	−0.5	−1.3 – 0.2	0.179
Moderate	−0.7	−1.3 – 0.0	0.049
Low	REF	-	-
**Relationship status**			
**Model 1**			
Yes	0.5	−0.1 – 1.1	0.096
No	REF	-	-
**Model 2**			
Yes	0.5	−0.1 – 1.0	0.101
No	REF	-	-
**Model 3**			
Yes	0.4	−0.1 – 1.0	0.138
No	REF	-	-
**Diet**			
**Model 1**			
Good	1.9	0.6 – 3.2	0.004
Intermediate	0.1	−0.5 – 0.7	0.704
Poor	REF	-	-
**Model 2**			
Good	2.0	0.7 – 3.4	0.003
Intermediate	0.1	−0.5 – 0.7	0.775
Poor	REF	-	-
**Model 3**			
Good	2.1	0.8 – 3.5	0.002
Intermediate	0.2	−0.4 – 0.7	0.608
Poor	REF	-	-
**Age**			
Model 1	0.1	0.08 – 0.14	< 0.001
Model 2	0.1	0.082 – 0.136	< 0.001
Model 3	0.1	0.08 – 0.14	< 0.001
**Time on ART at baseline**			
Model 1	−0.01	−0.02 – −0.002	0.010
Model 2	−0.01	−0.02 – −0.006	0.001
Model 3	−0.01	−0.02 – −0.001	0.024
**Model 1**			
PLWH on ART*Time (cat) at 12 months	0.3	0.1 – 0.6	0.010
PLWH on ART*Time (cat) at 24 months	0.2	−0.1 – 0.4	0.297
PLWH on ART*Time (cat) at 36 months	−0.1	−0.4 – 0.2	0.608
PLWH on ART*Time (cat) at 48 months	0.0	−0.5 – 0.4	0.908
PLWH on ART* Time (cat) at baseline visit	REF	-	-
**Model 2**			
PLWH, VL < 1000*Time (cat) at 12 months	0.3	0.1 – 0.6	0.012
PLWH, VL < 1000*Time (cat) at 24 months	0.2	−0.1 – 0.4	0.303
PLWH, VL < 1000*Time (cat) at 36 months	−0.1	−0.5 – 0.2	0.382
PLWH, VL < 1000*Time (cat) at 48 months	−0.3	−0.7 – 0.2	0.266
PLWH, VL < 1000* Time (cat) at baseline visit	REF	-	-
**Model 3**			
PLWH on stable ART *Time (cat) at 12 months	0.4	0.2 – 0.7	0.001
PLWH on stable ART*Time (cat) at 24 months	0.3	0.0 – 0.6	0.066
PLWH on stable ART*Time (cat) at 36 months	0.0	−0.3 – 0.4	0.798
PLWH on stable ART*Time (cat) at 48 months	−0.1	−0.6 – 0.5	0.787
PLWH on stable ART* Time (cat) at baseline visit	REF	-	-
PLWH, initiating treatment*Time (cat) at 12 months	0.5	0.1 – 1.0	0.030
PLWH, initiating treatment*Time (cat) at 24 months	−0.1	−0.6 – 0.5	0.788
PLWH, initiating treatment*Time (cat) at 36 months	−0.3	−0.9 – 0.3	0.334
PLWH, initiating treatment*Time (cat) at 48 months	−0.7	−1.6 – 0.1	0.097
PLWH, initiating treatment*Time (cat) at baseline visit	REF	-	-
Seroconverters *Time (cat) at 12 months	0.1	−0.7 – 0.9	0.836
Seroconverters *Time (cat) at 24 months	−0.2	−1.0 – 0.7	0.726
Seroconverters *Time (cat) at 36 months	−0.6	−1.6 – 0.4	0.261
Seroconverters *Time (cat) at 48 months	−0.9	−2.2 – 0.4	0.163
Seroconverters *Time (cat) at baseline visit	REF	-	
**Model 1**			
PLWH not ART*Time (cat) at 12 months	0.3	−0.3 – 0.8	0.361
PLWH not ART*Time (cat) at 24 months	0.2	−0.5 – 0.9	0.580
PLWH not ART*Time (cat) at 36 months	0.6	−0.3 – 1.5	0.226
PLWH not ART*Time (cat) at 48 months	0.2	−1.4 – 1.8	0.799
PLWH not ART*Time (cat) at baseline visit	REF	-	-
**Model 2**			
PLWH, VL ≥ 1000*Time (cat) at 12 months	−0.4	−0.9 – 0.1	0.090
PLWH, VL ≥ 1000*Time (cat) at 24 months	−0.4	−1.0 – 0.2	0.167
PLWH, VL ≥ 1000*Time (cat) at 36 months	−0.3	−1.0 – 0.3	0.308
PLWH, VL ≥ 1000*Time (cat) at 48 months	0.3	−0.7– 1.3	0.605
PLWH, VL ≥ 1000*Time (cat) at baseline visit	REF	-	-
**Model 3**			
PLWH either ART non-adherence or therapy resistance *Time (cat) at 12 months	0.7	−1.2 – −0.3	0.001
PLWH either ART non-adherence or therapy resistance *Time (cat) at 24 months	−0.5	−1.0 – 0.0	0.069
PLWH either ART non-adherence or therapy resistance *Time (cat) at 36 months	−1.0	−1.5 – −0.4	0.001
PLWH either ART non-adherence or therapy resistance *Time (cat) at 48 months	−0.5	−1.3 – 0.3	0.229
PLWH either ART non-adherence or therapy resistance *Time (cat) at baseline visit	REF	-	-
**Model 1, Model 2, Model 3**			
HIV-negative*Time (cat) at any month	REF	-	-
**Model 1**			
Female sex*Time (cat) at 12 months	−0.4	−0.6 – −0.1	0.003
Male sex*Time (cat) at 12 months	REF	-	-
**Model 2**			
Female sex*Time (cat) at 12 months	−0.3	−0.6 – −0.1	0.006
Male sex*Time (cat) at 12 months	REF	-	-
**Model 3**			
Female sex*Time (cat) at 12 months	−0.3	−0.6 – −0.1	0.006
Male sex*Time (cat) at 12 months	REF	-	-
**Model 1**			
Female sex*Time (cat) at 24 months	0.1	−0.2 – 0.4	0.408
Male sex*Time (cat) at 24 months	REF	-	-
**Model 2**			
Female sex*Time (cat) at 24 months	0.1	−0.1 – 0.4	0.350
Male sex*Time (cat) at 24 months	REF	-	-
**Model 3**			
Female sex*Time (cat) at 24 months	0.1	−0.1 – 0.4	0.345
Male sex*Time (cat) at 24 months	REF	-	-
**Model 1**			
Female sex*Time (cat) at 36 months	0.5 (-0.2 – 0.8)	-	0.001
Male sex*Time (cat) at 36 months	REF	-	-
**Model 2**			
Female sex*Time (cat) at 36 months	0.5 (0.2– 0.8)	-	0.001
Male sex*Time (cat) at 36 months	REF	-	-
**Model 3**			
Female sex*Time (cat) at 36 months	0.5 (0.2 – 0.8)	-	0.001
Male sex*Time (cat) at 36 months	REF	-	-
**Model 1**			
Female sex*Time (cat) at 48 months	0.5 (0.0– 0.9)	-	0.042
Male sex*Time (cat) at 48 months	REF	-	-
**Model 2**			
Female sex*Time (cat) at 48 months	0.5 (0.1– 0.9)	-	0.025
Male sex*Time (cat) at 48 months	REF	-	-
**Model 3**			
Female sex*Time (cat) at 48 months	0.5 (0.0 – 0.9)	-	0.037
Male sex*Time (cat) at 48 months	REF	-	-
**Model 1, Model 2, Model 3**			
Female sex*Time (cat) at baseline visit	REF	-	-
Male sex*Time (cat) at baseline visit	REF	-	-

CI, confidence interval; PLWH, people living with HIV; REF, reference; Cat, categorical; ART, antiretroviral therapy; ZAR, South African Rand; IPAQ, International Physical Activity Questionnaire.

* , Statistical significance was considered < 0.05.
